# Exercise Training and Natural Killer Cells in Cancer Survivors: Current Evidence and Research Gaps Based on a Systematic Review and Meta-analysis

**DOI:** 10.1186/s40798-022-00419-w

**Published:** 2022-03-04

**Authors:** Pedro L. Valenzuela, Gonzalo Saco-Ledo, Alejandro Santos-Lozano, Javier S. Morales, Adrián Castillo-García, Richard J. Simpson, Alejandro Lucia, Carmen Fiuza-Luces

**Affiliations:** 1 Faculty of Sport Sciences, Universidad Europea de Madrid, Madrid, Spain; 2 Physical Activity and Health Research Group (PaHerg), Instituto de Investigación Sanitaria Hospital, ‘12 de Octubre’ (‘imas12’), Centro de Actividades Ambulatorias (CAA), 7ª Planta, Bloque D, Av. de Córdoba s/n, 28041 Madrid, Spain; 3 I+HeALTH, Department of Health Sciences, European University Miguel de Cervantes, Valladolid, Spain; 4 MOVE-IT Research Group, Department of Physical Education, Faculty of Education Sciences, University of Cadiz, Cadiz, Spain; 5 Fissac – Physiology, Health and Physical Activity, Madrid, Spain; 6 School of Nutritional Sciences and Wellness, The University of Arizona, Tucson, AZ USA; 7 Department of Pediatrics, The University of Arizona, Tucson, AZ USA; 8 Department of Immunobiology, The University of Arizona, Tucson, AZ USA

**Keywords:** Immune system, Immunity, Training, Physical activity, Oncology, Immune function, Cytotoxic activity

## Abstract

**Background:**

Exercise training can positively impact the immune system and particularly natural killer (NK) cells, at least in healthy people. This effect would be of relevance in the context of cancer given the prominent role of these cells in antitumor immunity. In this systematic review and meta-analysis, we aimed to summarize current evidence on the effects of exercise training on the levels and function of NK cells in cancer survivors (i.e., from the time of diagnosis until the end of life).

**Methods:**

Relevant articles were searched in PubMed, Scopus, Web of Science and Cochrane Central Register of Controlled Trials (until January 11, 2022). Randomized controlled trials (RCT) of exercise training (i.e., non-acute) interventions *vs* usual care conducted in cancer survivors and assessing NK number and/or cytotoxic activity (NKCA) before and upon completion of the intervention were included. Methodological quality of the studies was assessed with the PEDro scale, and results were meta-analyzed using a random effects (Dersimoian and Laird) model.

**Results:**

Thirteen RCT including 459 participants (mean age ranging 11–63 years) met the inclusion criteria. Methodological quality of the studies was overall fair (median PEDro score = 5 out of 10). There was heterogeneity across studies regarding cancer types (breast cancer, non-small cell lung cancer and other solid tumors), treatment (e.g*.*, receiving *vs* having received chemotherapy), exercise modes (aerobic or resistance exercise, Tai Chi, Yoga) and duration (2–24 weeks). No consistent effects were observed for NK number in blood (mean difference [MD]: 1.47, 95% confidence interval [CI] − 0.35 to 3.29, *p* = 0.113) or NKCA as assessed in vitro (MD: − 0.02, 95%CI − 0.17 to 0.14, *p* = 0.834). However, mixed results existed across studies, and some could not be meta-analyzed due to lack of information or methodological heterogeneity.

**Conclusions:**

Current evidence does not support a significant effect of exercise training intervention on NK cells in blood or on their ‘static response’ (as assessed in vitro) in cancer survivors. Several methodological issues and research gaps are highlighted in this review, which should be considered in future studies to draw definite conclusions on this topic.

**Supplementary Information:**

The online version contains supplementary material available at 10.1186/s40798-022-00419-w.

## Key Points

- Physical exercise has the potential to positively impact the immune system and particularly natural killer (NK) cells. This would be of relevance in the context of cancer given the prominent role of these cells in antitumor immunity. Controversy exists, however, on the actual effects of exercise training on NK cells in patients with cancer.
- Although there is biological rationale for a potential benefit of exercise training on NK cells, current evidence does not support a significant effect in cancer survivors.
- Several potential sources of heterogeneity across studies, methodological issues and research gaps are present in the literature, which should be considered in future studies to draw definite conclusions on this topic.

## Background

Among its numerous health benefits, physical exercise seems to positively impact the immune system [1]. Evidence is particularly strong for a beneficial effect on natural killer (NK) cells [2], which participate in first line innate immune defense through their cytotoxic activity (NKCA) and release of effector cytokines such as interferon (IFN)γ or tumor necrosis factor (TNF)α [3]. There is indeed recent meta-analytical evidence that an acute session of exercise transiently increases (i.e., during 1 or 2 h post-exertion) NKCA in healthy individuals [4]. With regard to exercise training (i.e., frequent, systematic repetition of acute exercise sessions), although decreases or no changes have been reported in old people [5, 6] or in young female athletes (after intensive training) [5, 6], respectively, preclinical evidence in rodents indicates training-induced increases in NKCA [7, 8]. On the other hand, several cross-sectional studies have reported higher NKCA in endurance trained athletes (e.g*.*, cyclists, runners) than in their non-trained/sedentary peers [9–12], as well as in older people who performed physical activity regularly compared with their less active age-matched controls [13].

An eventual benefit associated with exercise training on NK cells might be of clinical relevance in the context of cancer given the important role of this lymphocyte subpopulation in antitumor immunity [14, 15]. Indeed, NK cells are constantly on high alert for malignant cell transformation and monitor target cells for surface expression of ligands for NK-activating receptors [16]. In fact, a high presence of NK cells in peripheral blood and particularly in the tumor microenvironment could be a positive prognostic factor in a variety of cancers [17]. A prospective study reported an inverse association between NKCA and cancer risk during an 11-year follow-up [18]. Recently, the percentage of circulating NK cells was positively associated with survival in patients with cancer [19]. Moreover, both the number of tumoral NK cells and NKCA have been positively associated with response to treatment and survival in patients with cancer [20–22]. On the other hand, a preclinical study by Pedersen et al. in NK cells showed that, although NKCA per se was unchanged in response to regular exercise (mouse voluntary wheel training), the exercise stimulus promoted NK cell infiltration in different types of tumors, with the level of NK cell infiltration inversely associated with tumor burden [23].

A similar relative increase in circulating NK cells has been observed in patients with cancer and in healthy individuals with no cancer history upon completion of an acute bout of moderate-intensity exercise, despite the former presenting lower absolute counts of these cells [24]. A comparable short-term increase in both NK cell number and expression of NKG2D (an activating NK cell receptor) has also been reported in patients with cancer and healthy controls for at least 24 h after running a half marathon [25]. More recently, although there were differences in perforin (a cytotoxic mediator released by NK cells to destroy target cells) and IFNγ expression that deserve further attention as they might have an influence on NKCA, Hanson et al. reported a similar increase in NK cells number and proportions immediately after a single session of moderate-intensity exercise (cycling) in prostate cancer survivors and healthy controls, with NK cell count returning to baseline levels at 24 h post-exertion [26]. According to the authors, the fact that NK cell proportions did not return to resting levels until 24 h of recovery suggests that consecutive sessions of acute exercise can be applied without adverse effects on the immune system during prostate cancer treatment. In this regard, however, there is controversy over the actual ‘chronic’ effects of exercise training on NK cells in patients with cancer (for a review see Zimmer et al. [27]). Some evidence suggests that exercise training interventions might induce a sustained increase in NKCA in survivors of breast cancer [28, 29], in patients with stomach cancer after curative surgery [30] or in children with cancer undergoing hematopoietic stem cell transplantation [31]. However, other studies have reported no improvement in NKCA after training intervention in patients with breast cancer [32] or in pediatric patients with solid tumors [33]. A systematic review (with no meta-analysis) of studies published until 2011 reported that there was strong evidence for an increase in NKCA with regular exercise in cancer patients, but not for a change in NK cell numbers [2]. More recently, a meta-analysis by Khosravi et al. found no significant effects of exercise training on NK cell proportions or NKCA in cancer survivors [34]. However, this is a rapidly evolving field and several studies that were not included in the meta-analysis by Khosravi et al. are now available that could yield new insights on the effects of exercise training on NK cells in cancer survivors [33, 35–37]. Thus, a new meta-analysis is needed to update medical evidence on this topic.

The purpose of the present systematic review and meta-analysis of randomized controlled trials (RCT) was to update the evidence on the effects of exercise training interventions on NK cells and NKCA in cancer survivors –– we adopted the definition of ‘cancer survivor’ proposed by the National Coalition for Cancer Survivorship, that is, from the time of diagnosis until the end of life (http://www.canceradvocacy.org). Research gaps and methodological issues are also discussed.

## Methods

The conduct and reporting of the current systematic review and meta-analysis conform to the Preferred Reporting Items for Systematic Reviews and Meta-analyses [38].

### Data Sources and Search Strategies

Two authors (CFL, GSL) independently conducted a systematic search (first by title and abstract, and then by full-text) in the electronic databases PubMed, Scopus, Web of Science and Cochrane Central Register of Controlled Trials (from inception to January 11, 2022) using the following search strategy: (exercise OR ‘physical activity’ OR training) AND (‘natural killer’ OR NK) AND (function OR activity OR cytotoxicity). A specific example of the systematic search is shown in Additional file 1. The search was supplemented by a manual review of reference lists from relevant publications to find additional studies on the subject.

### Study Selection

Studies written in English were eligible for inclusion if they met each of the following criteria: (1) used an RCT design; (2) were conducted in cancer survivors (during and/or after treatment); (3) included both an intervention group performing an exercise training program and a non-exercise control group; and (4) assessed NK cell number and/or NKCA in the two aforementioned groups before (baseline) and after the intervention period (post-intervention). Studies were excluded if they assessed the transient effects of a single training session (i.e., pre- versus post-acute exercise, with post-exertion assessments performed within a few hours after the session) on the aforementioned variables instead of the training effects per se (i.e., baseline versus post-intervention) or if they used a crossover design.

### Data Extraction

Two authors (CFL, GSL) independently extracted the following data from each study: number of participants within each group, participants’ (cancer type, treatment or post-treatment phase, sex, age) and exercise intervention characteristics (exercise modality, and length, frequency, duration and intensity of the training sessions), endpoints, assessment methods and results. Eventual disagreements were resolved through discussion with a third author (PLV). Data were extracted as mean and standard deviations (SD). A specific software (WebPlotDigitizer 4.2, San Francisco, CA) was used to extract those data provided as a figure. We contacted the authors of five studies where the necessary information was not available, but only the authors of one of them [28] reported the required information.

### Quality Assessment

Two authors (GSL, PLV) independently assessed the methodological quality of the included studies with the PEDro scale [39]. A 0–10 total score was determined by counting the number of criteria satisfied by each study. Study quality was rated as poor (PEDro score ≤ 3), fair (4–5) or high (> 5). All studies were used for data synthesis independently of their methodological quality. A third author (CFL) resolved any potential disagreement.

### Statistical Analysis

A random effects meta-analysis (DerSimonian and Laird method) was performed to assess the mean difference (MD, expressed as 95% confidence interval [CI]) between the intervention and control group in the change (post-intervention *minus* baseline) of:

1. NK number (CD56^+^/CD16^+^, CD3^−^ phenotype), expressed as relative values [percentage of total number of circulating lymphocytes or of peripheral blood mononuclear cells, PBMCs]).
2. NKCA (using the logarithm of the percentage lysis value for in vitro assays).

The weight assigned to each study included in the meta-analysis was defined by the SD of the variables and the sample size. A meta-analysis was only performed when a minimum of three studies analyzed the same outcome. If a given study reported results for different effector-to-target cell ratios, results were combined to create a single pair-wise comparison as explained elsewhere [40]. Beggs' test was used to determine the presence of publication bias, and the *I*^2^ statistic was used to assess heterogeneity across studies. Thus, *I*^2^ values ≥ 30% and < 50%, ≥ 50% and < 75%, or ≥ 75% were considered indicative of moderate, substantial or high heterogeneity across studies, respectively [40]. When high heterogeneity was present, sensitivity analyses were performed attending to variables such as cancer type, exercise modality or to whether participants were under treatment or had already completed treatment. Statistical analyses were performed using MIX 2.0 Pro for Excel software setting the level of significance at 0.05.

## Results

### Study Characteristics

**Fig. 1:**
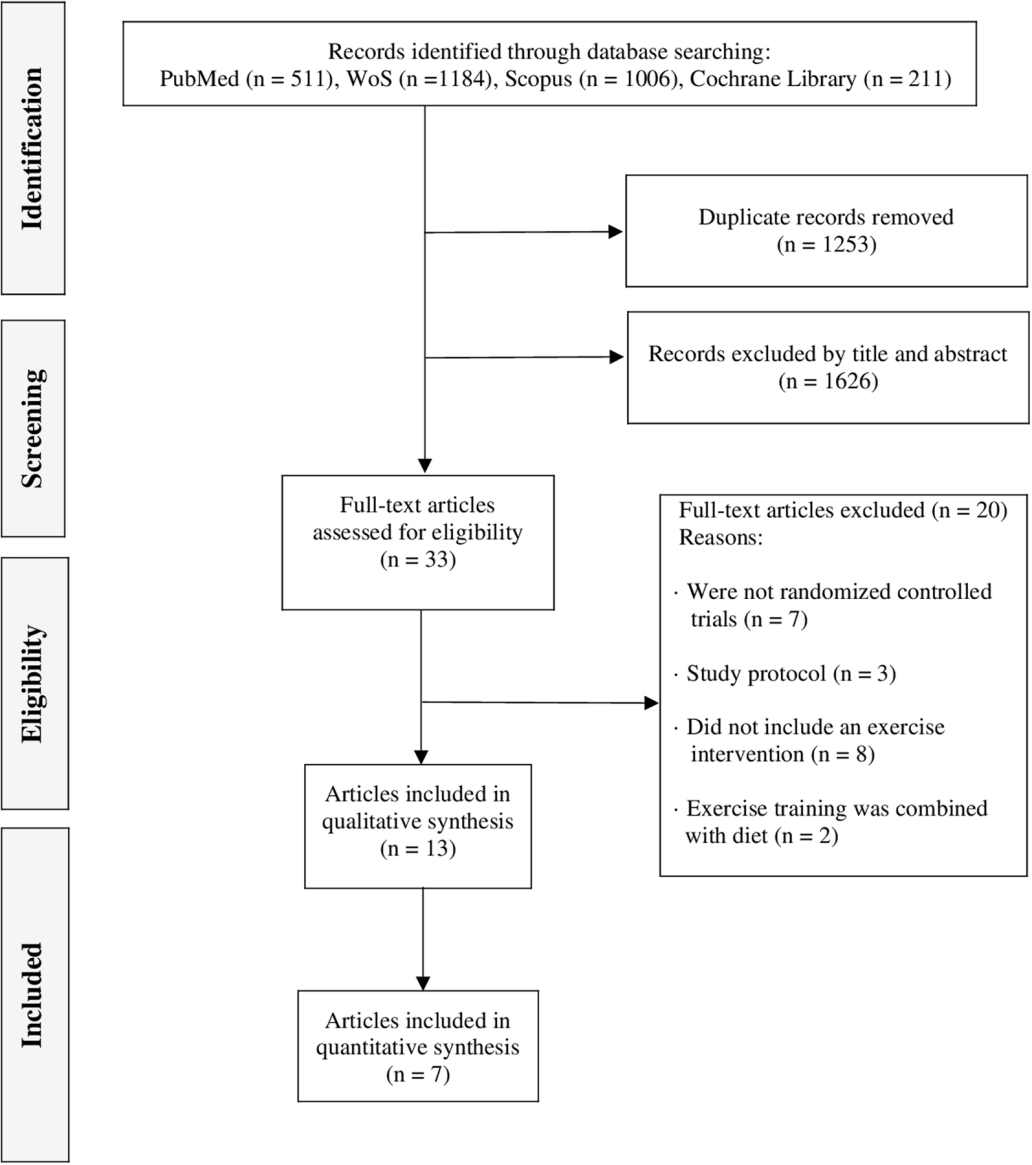
Flowchart of literature search. *WoS* Web of Science

From the retrieved studies, 13 were included in the systematic review (total *n* = 459 at baseline assessment, of whom 413 participants completed post-intervention assessments and were thus included in the analyses; mean age between 11 and 63 years) (Fig. 1) [28–30, 32, 33, 35–37, 41–45].

The characteristics of the included studies and their main results are summarized in Table 1. They were conducted in survivors of breast [28, 29, 32, 35–37, 45], stomach [30] and non-small cell lung cancer [41], or of several types of pediatric [33] or adult solid tumors [42–44]. Five studies analyzed the effects of exercise training in participants who were receiving chemotherapy [33, 37, 43–45], whereas in the remainder of studies participants had already completed treatment (surgery, radiotherapy and/or chemotherapy) before starting the exercise program [28–30, 32, 36, 41, 42].

**Table 1:** Main characteristics of the included studies

Study	Cancer and treatment	Study groups	Exercise training intervention	Technique	Variable	Results (main exercise training effects)
Fairey et al. [29]	Stage I to IIIB breast cancer	Exercise: *N* = 25 (24 analyzed) 59 ± 5 years	Modality: supervised aerobic training	Flow cytometry	Percentage of NK cells in PBMCs	↔ NK cells
	Completed surgery, radiotherapy and/or chemotherapy (14 ± 6 months prior to enrollment) with/without tamoxifen or anastrozole therapy		Length and frequency: 15 weeks, 3 days/week	^51^Cr release assay K562 as the target cell	NKCA in PBMCs: percentage of ^51^Cr release and PBMCs corrected to a per cell basis (lytic units/10^3^, number of effector cells required to cause 30% lysis of target cells)	↑ NKCA at all E:T ratios
		Control: *N* = 28; 58 ± 6 years	Duration: 15 – 35 min	E:T ratios = 50:1, 25:1, 12.5:1, 6.25:1, 3.125:1		
			Intensity: ~70–75% VO_2peak_			
Fiuza-Luces et al. [33]	Extracranial solid tumors	Exercise: *N* = 25 (9 analyzed) 11.0 ± 4.0 years	Modality: supervised aerobic and resistance training	Flow cytometry	Absolute number and percentage of NK cells, NK cell subsets (NK^bright^ and NK^dim^) and NK cells expressing different activating or inhibitory receptors in total blood	↔ NK cell
	During neoadjuvant chemotherapy	Control: *N* = 24 (11 analyzed) 12.0 ± 4.0 years	Length and frequency: the neoadjuvant chemotherapy period (19 ± 2 weeks), 3 days/week in gym sessions or 5 days/week in ward sessions			↔ NK cell receptors
				Europium-TDA release assay	NKCA in PBMCs: percentage of K562 lysis	↔ NKCA
			Duration: 30 min of aerobic training; 30 min of resistance training	K562 as the target cell		
				E:T ratios = 8:1. 4:1, 2:1, 1:1		
			Intensity: 60–70% HRmax (aerobic training); intensity progressively increased (resistance training)			
Glass et al. [44]	Solid tumors (mainly breast cancer)	Exercise: *N* = 26 (23 analyzed) 56 ± 10 years	Modality: supervised aerobic training	Flow cytometry	Percentage of NK cells in PBMCs	↔ NK cells
	During cytotoxic therapy and synthetic erythropoietin therapy		Length and frequency: 12 weeks, 3 days/week			
		Control: *N* = 29 (21 analyzed) 54 ± 11 years	Duration: 20–45 min			
			Intensity: 55–100% VO_2peak_			
Hagstrom et al. [28]	Stage I to IIIA breast cancer	Exercise: *N* = 20 (19 analyzed) 51.2 ± 8.5 years	Modality: supervised resistance training	Flow cytometry	Percentage of NK cells in total blood	↔ NK cells
	Completed surgery, radiotherapy and/or chemotherapy (11.5 months average prior to enrollment) with/without hormone therapy		Length and frequency: 16 weeks, 3 days/week	Flow cytometry	Percentage of functional markers (granzyme B and perforin) of NKCA in total blood	↔ granzyme B and perforin expression in NK cells
		Control: *N* = 19 (15 analyzed) 52.7 ± 9.4 years	Duration: 60 min	Flow cytometry	Percentage of NK cell intracellular cytokine (TNFɑ and IFNγ) production in total blood	↓ NK cell expression of TNFɑ
			Intensity: 8-RM			
Kaushik et al. [42]	Prostate cancer	Exercise *N* = 14 (12 analyzed) 56 (55–61) years	Modality: supervised Hatha yoga	Flow cytometry	Frequency and absolute number of NK cells and CD56^bright^ and CD56^dim^ NK cells in preactivated PBMCs	↔ NK cells
	Perioperative (radical prostatectomy) period	Control *N* = 15 (14 analyzed) 60 (59–61) Years	Length and frequency: 6 weeks preoperatively + 6 weeks starting 3–6 weeks postoperatively, 2 days/week	Flow cytometry	Cytokine response in NK cells from preactivated PBMCs	↑ Fc receptor III production and IFN-γ expression in NK cells
			Duration: 60 min			
			Intensity: adapted to patients’ comfort level			
Ligibel et al. [35]	Breast cancer	Exercise: *N* = 27 (14 analyzed) 52.3 ± 9.6 years	Modality: supervised and unsupervised aerobic/resistance training	Immunohistochemistry	Percentage of NK cells in tumor tissue	↔ NK cells
	Preoperative breast surgery		Length and frequency: mean of 29.3 days, 2 days/week			
		Control: *N* = 22 (11 analyzed) 53.1 ± 7.9 years	Duration: 180 min of aerobic training and 40 min of resistance training			
			Intensity: moderate			
Liu et al. [41]	Stages I to IIIB non-small cell lung cancer	Exercise: *N* = 16 (14 analyzed) 62.6 ± 8.4 years	Modality: supervised Tai Chi	Flow cytometry	Percentage of NK cells in total blood	↑ NK cells
	Post-surgery (≥ 2 years prior to enrollment)		Length and frequency: 16 weeks, 3 days/week	Cell viability assay	NKCA in PBMCs: tumor cell viability assay	↑ NKCA at 25:1 and 50:1 E:T ratios
		Control: *N* = 16 (13 analyzed) 60.5 ± 7.1 years	Duration: 60 min	A549 as the target cell		
			Intensity: moderate	E:T cell ratios = 50:1, 25:1, 12.5:1		
Mohamady et al. [36]	Breast cancer	Exercise: *N* = 20, 40–60 years	Modality: supervised and home-based training. Proprioceptive neuromuscular facilitation, resistance training and aerobic training	Flow cytometry	Number of NK cells in total blood	↑ NK cells
	Post-surgery	Control: *N* = 20, 40–60 years	Length and frequency: 12 weeks, twice a day, 3 – 7 days/week			
			Duration: 30 min			
			Intensity: moderate			
Na et al. [30]	Stomach cancer	Exercise: *N* = 17, 57.8 ± 12.1 years	Modality: supervised mobility exercises and resistance training (in bed) and supervised aerobic training (ambulatory setting)	^51^Cr release assay	NKCA in PBMCs: percentage of specific ^51^Cr release	↑ NKCA
	Post-surgery	Control: *N* = 18, 52.2 ± 10.3 years	Length and frequency: 2 weeks. Exercise in bed: 3 times a day. Ambulatory setting: 5 days/week, twice a day	K562 as the target cell		
			Duration: 30 min	E:T ratio = 50:1		
			Intensity: 60% HRmax (aerobic training); isometric (resistance training)			
Nieman et al. [32]	Breast cancer	Exercise: *N* = 8 (6 analyzed) 60.8 ± 4.0 years	Modality: supervised aerobic and resistance training	Flow cytometry	Percentage of NK cells in total blood	↔ NK cells
	Completed surgery, chemotherapy and/or radiotherapy within the previous 3.0 ± 1.2 years		Length and frequency: 8 weeks, 3 days/week	^51^Cr release assay	NKCA in PBMCs: percentage of ^51^Cr release	↔ NKCA
		Control: *N* = 8 (6 analyzed) 51.2 ± 4.7 years	Duration: 30 min of aerobic training and 30 min of resistance training	K562 as the target cell		
			Intensity: 75% HRmax (aerobic training); intensity progressively increased (resistance training)	E:T ratios = 40:1, 20:1		
Sagarra-Romero et al. [37]	Stage I and II breast cancer	Exercise: *N* = 11 (10 analyzed) 50.0 ± 5.5 years	Modality: supervised aerobic and resistance training	N/R	Percentage of NK cells in total blood	↔ NK cells
	Undergone surgery and receiving adjuvant chemotherapy		Length and frequency: 18–22 weeks, 3 days/week			
		Control: *N* = 11 (7 analyzed) 53.1 ± 6.8 years	Duration: 45 min; 20 min of aerobic training and 25 min of resistance training			
			Intensity: 60–70% VO_2peak_ (aerobic training) and N/R for resistance training			
Schmidt et al. [45]	Primary moderate- or high-risk breast cancer	Strength exercise: *N* = 21, 53 ± 12.6 years	Modality: supervised aerobic or resistance training	Flow cytometry	Absolute number of NK cells in total blood	↔ NK cells
	From the initiation of chemotherapy to the end of epirubicin or cyclophosphamide therapy	Aerobic exercise: *N* = 20, 56 ± 10.2 years	Length and frequency: 12 weeks, twice weekly			
			Duration: 60 min			
		Control: *N* = 26, 54 ± 11.2 years	Intensity: Borg scale of 11–14 (aerobic training) and 50% of the maximum weight with increments based on Borg scale (resistance training)			
Toffoli et al. [43]	Resectable colon (stage II/III) or breast cancer (stage I/II/III)	Exercise: *N* = 8 (4 for the expression of NK cell receptors) 55.1 ± 14.8 years	Modality: supervised aerobic and resistance training	Flow cytometry	NK cell subsets (NK^bright^ and NK^dim^) and NK cells expressing different activating or inhibitory receptors in total blood	↑ NKp46 (activating receptor) on CD56^dim^ CD16^+^ NK cells
	During neoadjuvant chemotherapy	Control: *N* = 6 (4 for the expression of NK cell receptors) 60.7 ± 7.6 years	Length and frequency: during the first 9–12 weeks or treatment, twice weekly	NK cell degranulation and cytotoxicity assay by flow cytometry	NK cell degranulation in monocyte-depleted PBMCs: percentage of CD107a^+^ NK cells	↔ NK cell degranulation
			Duration: 60 min	A431 as the target cell		
			Intensity: moderate to high	E:T ratio = 4:1	NKCA in monocyte-depleted PBMCs: relative percentage of cytotoxicity (of living tumor cells)	↔ NKCA

*Cr* chromium, *E:T* effector-to-target, *HRmax* maximum heart rate, *IFNγ* interferon gamma, *NK* natural killer, *NKCA*,NK cell cytotoxic activity, *N/R* not reported, *PBMCs* peripheral blood mononuclear cells, *RCT* randomized controlled trial, *RM* repetition maximum, *TDA* 2,2′:6′, 2″-terpyridine-6,6″-dicarboxylic acid, *TNFα* tumor necrosis factor-alpha, *VO*_*2peak*_ peak oxygen consumption. Symbols: ↑ increase, ↔ no change, ↓ decrease

Exercise training interventions lasted from 2 to 24 weeks and included 3–5 weekly sessions of ~ 20 to 90-min duration. Exercise sessions were supervised in most studies [28–30, 32, 33, 37, 41–45] although two included both supervised and non-supervised sessions [35, 36]. Different exercise modalities were used, including Tai Chi [41], Yoga [42], moderate-intensity continuous (‘aerobic’) training [29, 44, 45], resistance training [28, 45] or a combination thereof [30, 32, 33, 35–37, 43].

### Quality Assessment and Publication Bias

The quality of the included studies was overall fair (median PEDro score = 5 [range 4–8]; Table 2). Seven studies showed fair methodological quality [30, 32, 33, 36, 37, 42, 43], and six were deemed to have a high quality [28, 29, 35, 41, 44, 45].

**Table 2:** Methodological quality of the included studies

Authors (year)	Items											Total score*
	1	2	3	4	5	6	7	8	9	10	11	
Fairey et al. [29]	+	+	+	+	−	−	+	+	+	+	+	8
Fiuza-Luces et al. [33]	+	+	−	+	−	−	+	−	−	+	+	5
Glass et al. [44]	+	+	+	+	−	−	+	−	+	+	+	7
Hagstrom et al. [28]	+	+	−	+	−	−	+	+	+	+	+	7
Kaushik et al. [42]	+	+	−	+	−	−	−	+	−	+	+	5
Ligibel et al. [35]	+	+	−	+	−	−	+	−	+	+	+	6
Liu et al. [41]	+	+	+	+	−	−	−	+	−	+	+	6
Mohamady et al. [36]	+	+	−	−	−	−	−	+	−	+	+	4
Na et al. [30]	+	+	−	+	−	−	−	+	−	+	+	5
Nieman et al. [32]	+	+	−	+	−	−	−	+	−	+	+	5
Sagarra-Romero et al. [37]	+	+	−	+	−	−	−	−	−	+	+	4
Schmidt et al. [45]	+	+	+	+	−	−	+	−	−	+	+	6
Toffoli et al. [43]	+	+	−	+	−	−	−	−	−	+	+	4

Column numbers correspond to the following criteria on the PEDro scale: 1—eligibility criteria were specified; 2—subjects were randomly allocated to groups; 3—allocation was concealed; 4—groups were similar at baseline; 5—subjects were blinded; 6—therapists who administered the treatment were blinded; 7—assessors were blinded; 8—measures of key; outcomes were obtained from more than 85% of subjects; 9—data were analyzed by intention to treat; 10—statistical comparisons between groups were conducted; 11—point measures and measures of variability were provided. +indicates the criterion was clearly satisfied; −indicates that it was not. * A total score out of 10 is determined from a number of criteria that are satisfied, except that scale item 1 is not used to generate the total score

### Exercise Training Effects on NK Number

Except for Na et al. [30], all the included studies assessed exercise training effects on NK number, with this variable expressed as either absolute [33, 36, 42, 45] or relative values [28, 29, 32, 33, 35, 37, 41, 44]. Only one study assessed NK number within the tumor [35], whereas the rest analyzed peripheral blood samples. Ten studies used flow cytometry [28, 29, 32, 33, 36, 41–45], one study used a multiplex fluorescence immunohistochemistry assay on formalin-fixed tissue samples [35], and one did not specify the technique used [37].

Most studies [28, 29, 32, 33, 35, 37, 42, 44, 45] found no significant effects of exercise training on NK cell number, but two [36, 41] reported a significant increase in the relative and absolute level of these cells in peripheral blood with exercise training, respectively, and another report [45] found a significant reduction in the number of NK cells from baseline to post-intervention with endurance training—albeit no comparison with the control group was reported.

**Fig. 2:**
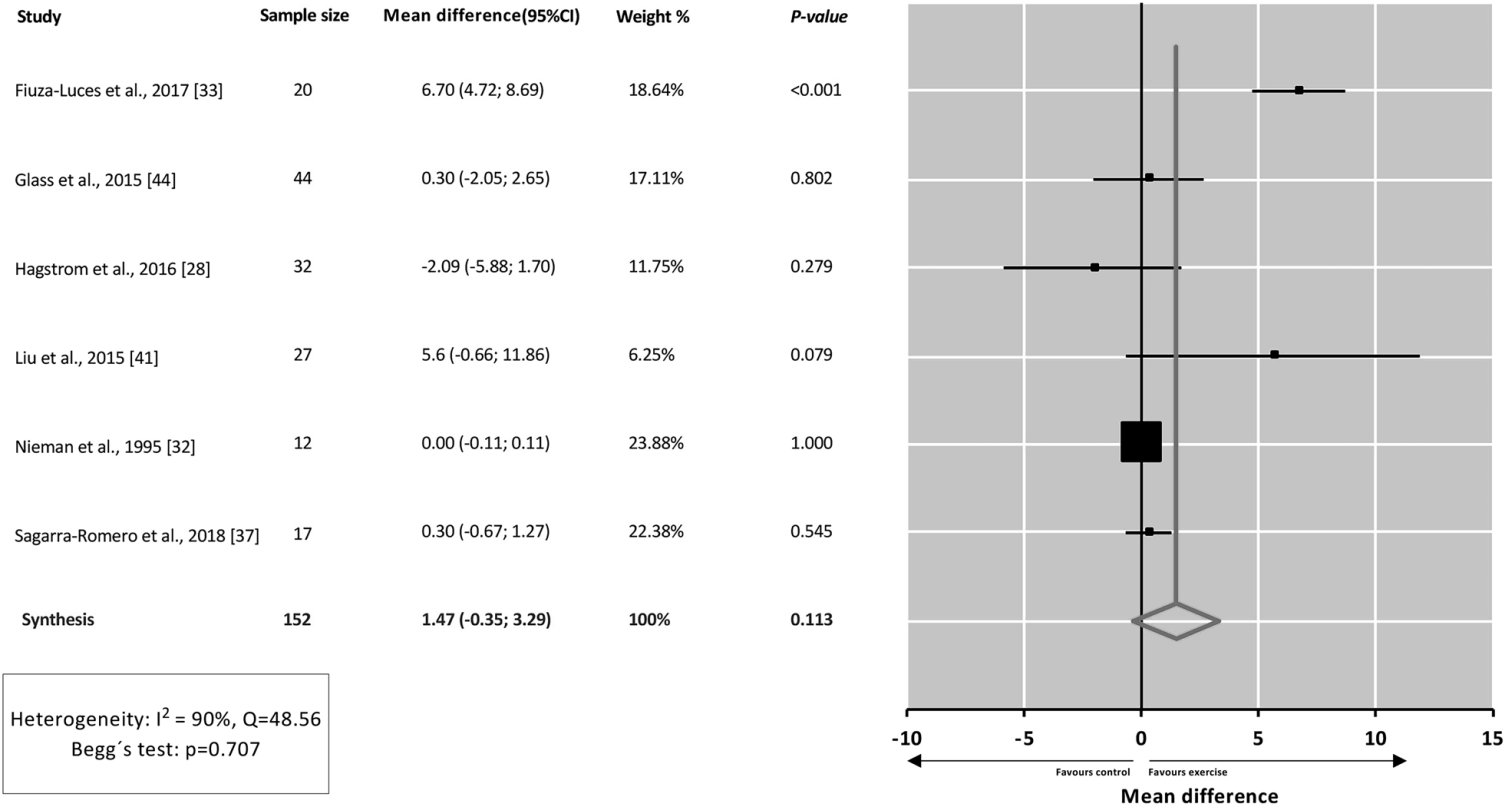
Effects of exercise training interventions on natural killer cell number (expressed as percentage of total number of circulating lymphocytes or peripheral blood mononuclear cells). *CI* confidence interval, *MD* mean difference

Six studies could be meta-analyzed [28, 32, 33, 37, 41, 44], and results showed no significant effects of exercise training on NK cell number in peripheral blood expressed in relative values (MD = 1.47; 95% CI − 0.35 to 3.29; *p* = 0.113) (Fig. 2), with no signs of bias (*p* = 0.707) but with high heterogeneity between studies (*Q* = 48.56, *I*^2^ = 90%). Of note, several studies [29, 35, 36, 42, 43, 45] were not pooled in the analyses for the reasons that are explained below. In the study by Schmidt et al. [45], the NK cell number was reported in absolute units. Ligibel et al. [35] studied NK cells within the tumor. Toffoli et al. [43] analyzed the expression of NK cell receptors and NK cell subsets without actually reporting the number of NK cells. Kaushik et al. [42] determined the number of NK cells after PBMC activation. Finally, two other studies [29, 36] were not included because they did not report the necessary data.

Sensitivity analyses could be performed attending to (1) type of cancer (i.e., breast cancer alone), (2) exercise modality (i.e., combined [aerobic and resistance] training) or (3) whether participants were under treatment or had already completed treatment, with lack of statistical significance remaining (Additional file 2). The results of each study with the relevant effect sizes (as reported by each study in question, e.g., post-intervention values or changes in values with results of statistical significance tests, if available) are shown in Additional file 3.

### Exercise Training Effects on NK Cytotoxicity

Three studies assessed NKCA with a standard in vitro cytotoxicity assay [29, 30, 32] which tests the ability of PBMCs to lyse ^51^Cr radiolabeled tumor cell targets (leukemia cell line K562 in most cases) in different effector-to-target cell ratios. However, one study utilized non-radioactive agents [33], loading the target cells with bis[acetoxymethyl] 2,2′:6′,2″-terpyridine-6,6″-dicarboxylate (BATDA). Liu et al. [41] tested the cytotoxicity of PBMCs against a human cell line of non-small cell lung cancer (A549) and tumor cell viability was also determined. Hagstrom et al. [28] assessed NKCA through the analysis of functional markers (such as granzyme B and perforin) or NK cell intracellular cytokine (such as TNFɑ and IFNγ) production. These authors found a significant reduction in the expression of TNFα in NK cells after resistance exercise training (but not after the control intervention), which was considered to reflect a beneficial adaptation (i.e., reduced inflammation). Toffoli et al. [43] analyzed NK cell degranulation and cytotoxicity in monocyte-depleted and activated (using interleukin (IL)-2 and IL-15) PBMCs against epidermoid carcinoma A431 cells using flow cytometry and found nonsignificant between-group differences.

Of these five studies using NK cytotoxicity assays, three reported a beneficial effect of exercise training on NKCA [29, 30, 41] and two reported no significant effects [32, 33]. Three studies could be meta-analyzed [29, 32, 33], with pooled analysis showing no significant effects on NKCA (− 0.02, 95%CI − 0.17 to 0.14, *p* = 0.834). There were no signs of bias (*p* = 0.602), but a high heterogeneity between studies was found (*Q* = 35.56, *I*^2^ = 94%). The study by Na et al. [30] did not provide the necessary data to be meta-analyzed. No sub-analyses could be performed due to the low number of studies available (*n* = 3). The results of each study are shown in Additional file 3.

## Discussion

The main result of our meta-analysis is the lack of a significant effect of exercise training on NK cell number or NKCA in cancer survivors, although there was high heterogeneity between studies for the two outcomes. Both increasing [36, 41] or decreasing effects [45] on NK cell number have been reported after exercise training in cancer survivors, but with most studies finding no significant effects [28, 29, 32, 33, 35, 37, 44, 42, 45]. Similarly, although some studies have reported an increasing effect of exercise training on NKCA in cancer survivors [29, 30, 41], others found no effects [32, 33]. Thus, despite the solid meta-analytical evidence—with data not only of healthy people, but also of cancer patients [24–26] included in the pooled analysis—that was recently reported for a transient increasing effect (within 1 or 2 h) of acute exercise on NKCA (which in fact seems independent of NK cell number) [4], current evidence does not support a significant ‘chronic’ or sustained effect of exercise training on the number or function of NK cells in cancer survivors—with NK cell function assessed in vitro, that is, ‘static’ response. Further research is needed to confirm the present results as well as to determine whether exercise training might induce significant effects on NK cells when assessed in vivo (‘dynamic’ NK cell function), as reported in preclinical research (e.g*.*, increased responsiveness to chemotactic signals and ability to infiltrate tumors) [23].

### Biological Underpinnings

The mechanisms by which regular physical exercise might eventually exert a sustainable effect on the mobilization of NK cells into the blood or on the function of these cells remain to be clearly elucidated as well as whether causative mechanisms might differ (or not) between cancer survivors or individuals with no history of cancer. Indeed, although transient increases in the myokine interleukin-6 and in epinephrine (and subsequent binding of these two molecules to NK cell receptors) [23, 46, 47] or in tumor blood perfusion [48, 49] have been postulated to stimulate NK cell mobilization into the blood and/or tumors during (or shortly after) acute exercise, these effects cannot explain an eventual sustained effect of repeated exercise sessions (i.e., exercise training). Dias et al. [50] found that 18 weeks of aerobic endurance training changed the expression of 211 gene transcripts in PBMCs that are known to be involved in cell cycle regulation, proliferation and development of immune cells, and a study comparing young endurance-trained athletes and non-athletic controls identified 72 candidate transcripts in PBMCs involved in encoding ribosomal proteins and oxidative phosphorylation [51]. PBMCs are, however, a heterogeneous mix of immune cells and changes in gene expression over time may be driven by alterations in immune cell proportions and not necessarily in NK cells per se. A recent study showed that a 12-week resistance training intervention had negligible effects on the NK cell transcriptome [52]. Slight increases were found for some candidate gene transcripts, of which only one of them, ten–eleven translocation methylcytosine dioxygenase 1 (involved in DNA demethylation) has a relevant role with regard to NK cell function. In a recent NK cell proteome study, we found that several proteins were upregulated after 6 weeks' moderate-intensity aerobic training, of which two (phosphoinositide-3-kinase regulatory subunit 1 [PIK3R1] and nucleoporin 88 kDa [NUP88]) have well documented roles in immune function [53]. Phosphoinositide-3-kinase (PIK3) signaling plays an important role in multiple key aspects of NK cell biology, including development/maturation, homing, priming and function of these cells [54], whereas NUP88 selectively mediates the nucleocytoplasmic transport of nuclear factor kappa-light-chain-enhancer of activated B cells (commonly known as NF-kB), an ubiquitous transcription factor involved in immune responses, apoptosis and cancer [55].

### Potential Sources of Heterogeneity Across Studies

The present meta-analysis revealed a high statistical heterogeneity between studies for the effects of exercise training on NK cell number (*I*^2^ = 90%) and NKCA (*I*^2^ = 80%) among cancer survivors. There were indeed differences between the studies we included in our analyses regarding participants’ age, cancer type and stage, or treatment (particularly, under treatment *vs* post-treatment at the time the study was done). However, in the present meta-analysis results of NK cell numbers remained nonsignificant even in sub-analyses attending to potential sources of heterogeneity such as type of tumor (breast cancer), exercise modality (combined training) and phase of cancer (during or after treatment). It must be noted, nonetheless, that the low number of studies available might have precluded us from finding statistically significant results. The fact that no other sub-analyses could be performed—and in fact no sub-analyses could be done at all for NKCA—might have also confounded our results. The heterogeneity found for the type (aerobic or resistance training, Tai Chi or Yoga) and intensity of exercise training interventions could also potentially contribute to the diversity of findings across studies. It could be hypothesized that exercise performed at higher intensities could elicit larger effects on NK cell mobilization and NKCA. In this regard, only three of the included studies in this review applied moderate to high exercise intensities (e.g*.*, > 70% of 1-repetition maximum or of maximum oxygen uptake [VO_2max_] for resistance or aerobic exercise, respectively) [28, 33, 44]. Moreover, a recent study reported no differences between the effects of moderate- or high-intensity exercise training, respectively, on NKCA in healthy adults (although the latter induced greater increases in NK cell number) [53], and another study recently reported no differences between moderate- and high-intensity exercise on NK cell number or function in women at high risk for breast cancer [56]. Differences in participants’ fitness status can also potentially influence NK cell adaptations to exercise training, and indeed, a recent study reported a negative correlation between baseline cardiorespiratory fitness and the change in NK cell function after a high-intensity training intervention, thereby suggesting that regular exercise might provide greater benefits in individuals with the lowest fitness level [56].

### Methodological Limitations and Future Lines of Research

**Fig. 3:**
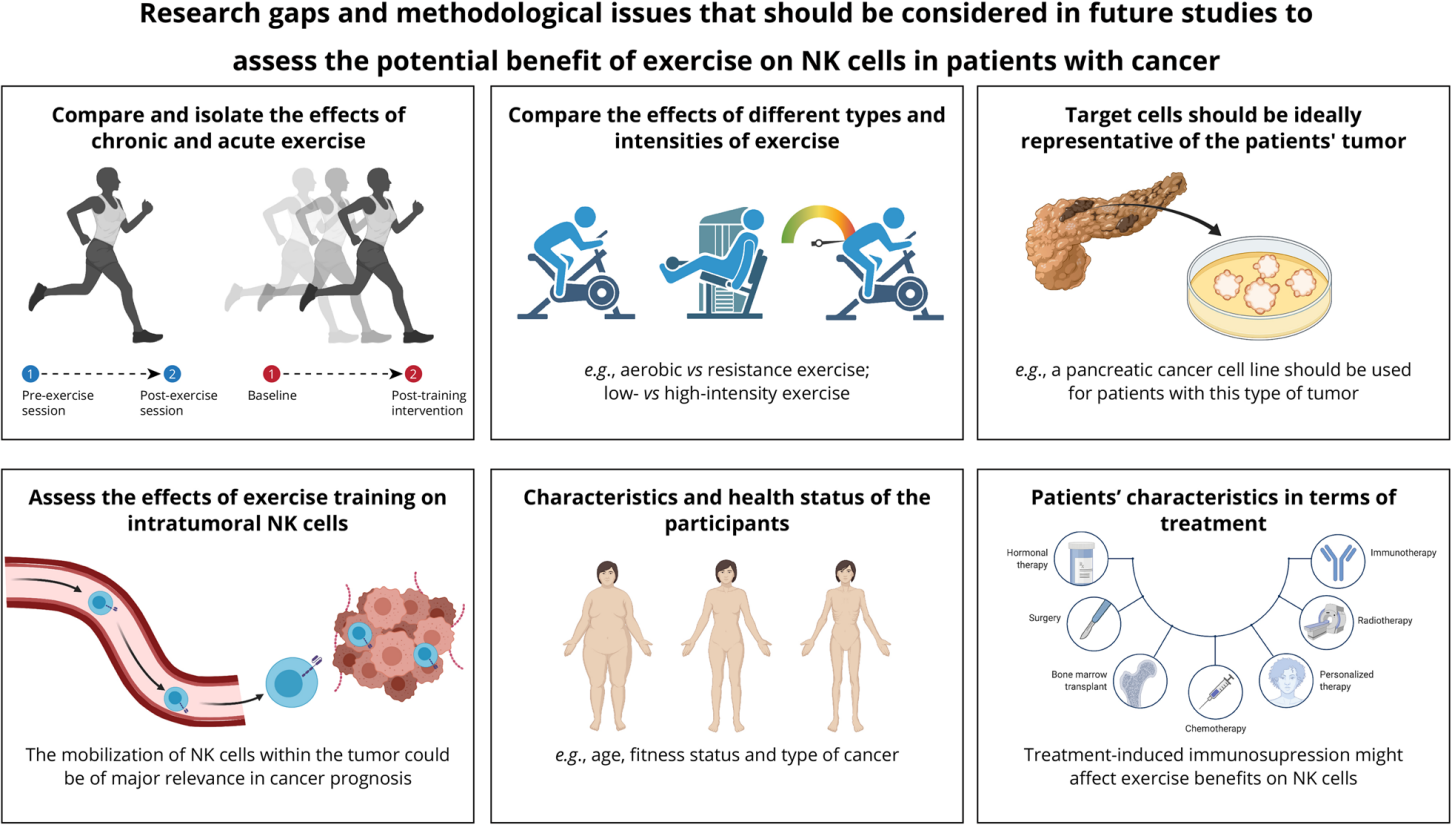
Research gaps and methodological issues that should be considered in future studies assessing the potential effects of exercise on natural killer (NK) cells in the context of cancer

Some methodological limitations must be noted (see also Fig. 3 for a summary), as they could have confounded, at least partly, the results of the studies we assessed. In order to isolate the potential long-term, sustainable effects of exercise training (i.e., baseline *vs* post-training intervention) from the transient effects of a single exercise bout (i.e., pre- versus post-acute exercise), NK cell number and NKCA should be ideally assessed after a washout period of ≥ 24 h after the last exercise training session—thereby allowing accounting for the potential confounding effects of the ‘exercise window’ [26]. However, eight studies [28, 30, 32, 36, 37, 42, 43, 45] did not report the time elapsed since the last training session. In the other studies, the participants were instructed not to exercise for at least 24 [41] or 48 h [29, 33, 44]. Moreover, most included studies assessed NK cell numbers and NKCA in peripheral blood, but to our knowledge only one study to date has assessed the effects of exercise training on intratumoral NK cells in humans [35]. In this effect, promoting the mobilization of NK cells within the tumor would be of major relevance given their important role in cancer prognosis [20–22]. However, Ligibel et al. [35] found no effects of exercise training on intratumoral NK cell mobilization, although the analyses of tumor gene showed an upregulation of pathways related to NKCA. Further research is therefore needed to confirm the effects of exercise training on NK cells not only in peripheral blood but also within the tumor. Another issue of potential clinical relevance is the type of target tumor cells used for assessing NKCA. Target cells should be ideally representative of the patients' tumor in question (e.g*.*, a breast cancer cell line should be used for patients with this type of tumor), whereas the commonly used leukemia cell line K562 might not represent the best option, except for secondary tumors.

More research is also needed focusing on the potential effects of exercise training on activating/inhibiting NK cell receptors. Only two of the studies included in the present meta-analysis assessed this question [33]. Fiuza-Luces et al. [33] quantified 13 NK cell subset receptors (including several killer Ig-like receptors, abbreviated as ‘KIR’) in children with solid tumors before and after neoadjuvant treatment. Although statistical significance was not reached, there was a trend toward an exercise training effect for NK cells expressing the receptor KIR2DS4, with their numbers remaining stable in the exercise group but increasing in controls. This finding might add further controversy to the issue as KIR2SD4 might play an activator, rather than an inhibitory role: notably obesity, a condition associated with an increased risk for several cancer types and an altered phenotype and functionality of NK cells, is linked to a decreased KIR2DS4 expression in NK cells [57]. On the other hand, it remains to be known whether potential exercise training effects on NK cells are of actual clinical relevance for cancer survivors. In this effect, except for the study by Na et al. (with these authors reporting that the exercise training intervention had a significant increasing effect on NKCA in stomach cancer patients after surgery, but with NKCA changes not associated with the occurrence of lymph node metastases) [30], none of the RCTs included in our meta-analysis assessed the association between an eventual exercise training-induced effect on NK cells and major clinical outcomes (e.g*.*, not only metastases but also survival or tumor recurrence). However, it must also be noted that Toffoli et al. [43] found a higher expression of the activating receptor NKp46 on CD56dim CD16 + NK cells in the exercise training group compared to the control group, a finding that might suggest a beneficial effect of exercise training on NK cell activity.

It also remains to be elucidated whether the effects of exercise training on NK cells are similar (or not) in cancer survivors and in people with no cancer history. In this regard, cancer and its treatments are frequently associated with pronounced immune deficiency [58, 59], and individuals diagnosed with cancer exhibit lower systemic NKCA than those with no cancer history [60, 61]. Thus, it could be hypothesized that due to cancer-induced immunosuppression, exercise training effects on cancer patients might be less remarkable than those reported in healthy individuals [53].

### Limitations of the Present Study

Some limitations of the present meta-analysis should also be acknowledged. First, the study was not preregistered, which might be considered a potential source of bias [62]. The low number of available studies and the differences found between them in terms of participants’ and interventions’ characteristics precluded us from performing sub-analyses attending to several variables that might have confounded our results. Further research is therefore needed to compare the effects of different modalities (with regard to exercise type, intensity and duration) of training interventions on NK cells in cancer survivors, as well as to determine whether effects could be dependent on the patients’ characteristics. Finally, data from some studies could not be obtained due to lack of information needed for analyses (despite contacting the corresponding authors) or to the use of different assessment techniques. The fact that these studies could not be included in the quantitative analyses might be regarded as a potential bias.

## Conclusions

Although there is biological rationale for a potential exercise-induced benefit on immune function and particularly on NK cells, further research is needed to elucidate whether regular exercise training can exert sustainable increases in the number and/or function of NK cells in cancer survivors, with current evidence not supporting a significant effect. The research gaps and methodological issues highlighted here should be considered in future studies to draw definite conclusions on this topic.

## Supplementary Information

**Additional file 1**. Search strategy.

**Additional file 2**. Sub-analyses on the effects of exercise training intervention on natural killer cell number.

**Additional file 3**. Results reported by each individual study.

## Data Availability

The datasets used and/or analyzed during the current study are available from the corresponding author on reasonable request.

## Abbreviations

BATDA: Bis[acetoxymethyl] 2,2′:6′,2″-terpyridine-6,6″-dicarboxylate
CI: Confidence interval
IFN: Interferon
IL: Interleukin
KIR: Killer Ig-like receptor
MD: Mean difference
NF-kB: Nuclear factor kappa-light-chain-enhancer of activated B cells
NK: Natural killer
NKCA: Natural killer cytotoxic activity
NUP88: Nucleoporin 88 kDa
PBMCs: Peripheral blood mononuclear cells
PIK3: Phosphoinositide-3-kinase
PIK3R1: Phosphoinositide-3-kinase regulatory subunit 1
RCT: Randomized controlled trials
SD: Standard deviation
TNF: Tumor necrosis factor
VO_2max_: Maximum oxygen uptake
