# Supplementary material for: Exercise Training and Natural Killer Cells in Cancer Survivors: Current Evidence and Research Gaps Based on a Systematic Review and Meta-analysis
Source: Sports Med Open. 2022 Mar 4;8:36. doi: 10.1186/s40798-022-00419-w (PMC8897541; doi:10.1186/s40798-022-00419-w)
Supplement: Supplementary file 1 — Additional file 1. Search strategy. [file 40798_2022_419_MOESM1_ESM.docx]

**Additional file 1**. **Search Strategy.**

| **1.** | exercise [tw] |
| --- | --- |
| **2.** | "physical activity" [tw] |
| **3.** | training [tw] |
| **4.** | OR 2 OR 3 |
| **5.** | "natural killer" [tw] |
| **6.** | NK [tw] |
| **7** | OR 6 |
| **8.** | function [tw] |
| **9.** | activity [tw] |
| **10.** | cytotoxicity [tw] |
| **11.** | OR 9 OR 10 |
| **12.** | 3 AND 6 |

- Filters: Tittle and abstract (PubMed); title, abstract and keyword (Scopus), Topic (Web of Science); title, abstract and keyword (Cochrane Central Register of Controlled Trials)

- PubMed, Scopus, Web of Science and Cochrane Central Register of Controlled Trials (from inception to January 11^th^, 2022).
