# Supplementary material for: Exercise Training and Natural Killer Cells in Cancer Survivors: Current Evidence and Research Gaps Based on a Systematic Review and Meta-analysis
Source: Sports Med Open. 2022 Mar 4;8:36. doi: 10.1186/s40798-022-00419-w (PMC8897541; doi:10.1186/s40798-022-00419-w)
Supplement: Supplementary file 2 — Additional file 2. Sub-analyses on the effects of exercise training intervention on natural killer cell number. [file 40798_2022_419_MOESM2_ESM.docx]

| Sub-analysis | Studies  (participants) | MD  (95%CI) | *p*-value | I^2^ | Begg’s *p*-value |
| --- | --- | --- | --- | --- | --- |
| **NK cells (%)** | | | | | |
| Breast cancer [28, 32, 37] | 3 (*N* = 61) | 0.002 (-0.11, 0.12) | 0.970 | 0% | 0.602 |
| Combined (aerobic + resistance) training [32, 33, 37] | 3 (*N* = 49) | 2.07 (-0.37, 4.50) | 0.096 | 96% | 0.017 |
| Finished treatment [28, 32, 41] | 3 (*N* = 71) | 0.209 (-2.44, 2.59) | 0.877 | 53% | 0.604 |
| Did not finish treatment [33, 37, 44] | 3 (*N* = 81) | 2.41 (-1.65, 6.47) | 0.244 | 86% | 0.580 |

**Additional file 2.** Sub-analyses on the effects of exercise training intervention on natural killer (NK) cell number (expressed as percentage of total number of circulating lymphocytes or peripheral blood mononuclear cells).

Abbreviations: CI, confidence interval; MD, mean difference.
