# Supplementary material for: Exercise Training and Natural Killer Cells in Cancer Survivors: Current Evidence and Research Gaps Based on a Systematic Review and Meta-analysis
Source: Sports Med Open. 2022 Mar 4;8:36. doi: 10.1186/s40798-022-00419-w (PMC8897541; doi:10.1186/s40798-022-00419-w)
Supplement: Supplementary file 3 — Additional file 3. Results reported by each individual study. [file 40798_2022_419_MOESM3_ESM.docx]

|  | Baseline | Postintervention | P-value |
| --- | --- | --- | --- |
| 50:1 E:T ratio, % lysis |  |  |  |
| Exercise | 55.5 (12.1) | 61.4 (9.8) | 0.039 |
| Control | 58.0 (12.9) | 56.4 (10.5) |  |
| 25:1 E:T ratio, % lysis |  |  |  |
| Exercise | 44.2 (12.8) | 49.8 (8.3) | 0.024 |
| Control | 45.3 (12.1) | 44.0 (11.3) |  |
| 12.5:1 E:T ratio, % lysis |  |  |  |
| Exercise | 36.2 (10.6) | 41.2 (8.4) |  |
| Control | 32.2 (10.1) | 33.8 (10.7) | 0.041 |
| 6.25:1 E:T ratio, % lysis |  |  |  |
| Exercise | 21.7 (9.0) | 27.7 (10.1) |  |
| Control | 18.9 (9.4) | 19.8 (9.2) | 0.022 |
| 3.125:1 E:T ratio, % lysis |  |  |  |
| Exercise | 7.2 (5.1) | 12.4 (6.6) |  |
| Control | 5.8 (4.5) | 5.7 (4.2) | <0.001 |
| Total lytic units |  |  |  |
| Exercise | 11.98 (6.76) | 8.60 (3.40) |  |
| Control | 12.72 (8.19) | 11.68 (6.00) | 0.035 |

**Farey et al. [29]**

**Fiuza-Luces et al. [33]**

|  |  | Baseline | Postintervention | p-value |
| --- | --- | --- | --- | --- |
| Ratio 8:1 | Exercise | 31.8 (5.0) | 22.6 (4.7) | 0.795 |
|  | Control | 19.4 (4.0) | 9.7 (3.6) |  |
|  |  |  |  |  |
| Ratio 4:1 | Exercise | 21.9 (4.0) | 17.5 (4.0) | 0.922 |
|  | Control | 15.4 (3.1) | 13.0 (3.2) |  |
|  |  |  |  |  |
| Ratio 2:1 | Exercise | 17.9 (3.9) | 14.0 (3.1) | 0.867 |
|  | Control | 12.5 (2.4) | 11.2 (3.2) |  |
|  |  |  |  |  |
| Ratio 1:1 | Exercise | 16.1 (4.0) | 11.1 (2.7) | 0.752 |
|  | Control | 10.5 (1.3) | 10.5 (3.3) |  |
|  |  |  |  |  |
| NK cells | Exercise | 7.1 (2.0) | 16.9 (2.6) | 0.894 |
| % | Control | 11.3 (2.5) | 14.5 (2.2) |  |
|  |  |  |  |  |
| 1000/μL | Exercise | 0.5 (0.1) | 1.1 (0.8) | 0.966 |
|  | Control | 1.0 (0.3) | 1.0 (0.4) |  |

**Glass et al. [44]**

| NK cells | Baseline | Postintervention | P-value |
| --- | --- | --- | --- |
| CD16^+^ / CD56^+^ (NK cells) |  |  |  |
| Exercise | 8.2% (4.4) | 9.7% (8.4) | 0.929 |
| Control | 8.8% (5.5) | 9.8% (8.0) |  |

**Hagstrom et al. [28]**

|  | Baseline | Postintervention | p-value |
| --- | --- | --- | --- |
| NK (%) |  |  | 0.94 |
| Exercise | 10.4 ± 4.9 | 9.36 (4.08) |  |
| Control | 9.5 ± 6.2 | 10.55 (6.67) |  |

**Kaushik et al. [42]**

These authors determined the number of NK cells after PBMC activation, and therefore are not valid for the purposes of the present meta-analysis.

**Ligibel et al. [35]**

|  | Baseline | Fold change | P value |
| --- | --- | --- | --- |
| CD 56+ (%) |  |  |  |
| Exercise | 1,86 (2.15) | 1.33 (2.34) | 0.95 |
| Control | 2,14 (3.94) | 0.90 (0.81) | 0.53 |

**Liu et al. [41]**

|  | Baseline | Postintervention | P-value |
| --- | --- | --- | --- |
| NKCA |  |  |  |
| 50:1 E:T ratio |  |  | < 0.05 |
| Exercise | 1.04 ± 0.19 | 0.51 ± 0.15 |  |
| Control | 1.04 ± 0.33 | 0.99 ± 0.25 |  |
|  |  |  |  |
| 25:1 E:T ratio |  |  | < 0.05 |
| Exercise | 0.85 ± 0.16 | 0.51 ± 0.13 |  |
| Control | 0.85 ± 0.39 | 0.76 ± 0.22 |  |
|  |  |  |  |
| 12.5:1 E:T ratio |  |  | > 0.05 |
| Exercise | 0.53 ± 0.15 | 0.52 ± 0.20 |  |
| Control | 0.66 ± 0.20 | 0.63 ± 0.20 |  |
|  |  |  |  |
| NK (%) |  |  | < 0.05 |
| Exercise | 22.66 ± 8.23 | 27.94 ± 10.3 |  |
| Control | 20.49 ± 6.82 | 20.17 ± 7.35 |  |

**Mohamadi et al. [36]**

Data not reported

**Na et al. [30]**

| E:T 5O:1 | Baseline | Postintervention |
| --- | --- | --- |
| Exercise | 16.2% (11.4) | 27.9% |
| Control | 19.7% (19.6) | 13.3% |
|  | (p > .05). | (p< .05). |

**Nieman et al. [32]**

|  |  | Baseline | Postintervention | P-value |
| --- | --- | --- | --- | --- |
| NK cells |  |  |  |  |
|  | Exercise | 0.3 ± 0.1 | 0.3 ± 0.1 | 0.99 |
|  | Control | 0.2 ± 0.1 | 0.2 ± 0.1 |  |
| NKCA, % lysis |  |  |  |  |
| E:T 40: 1 | Exercise | 39.7±6.3 | 44.3±4.6 | 0.15 |
|  | Control | 24.6±2.6 | 38.0± 3.3 |  |
|  |  |  |  |  |
| E:T 20:1 | Exercise | 28.9±5.8 | 41.3±4.8 | 0.75 |
|  | Control | 16.0±2.0 | 30.7± 3.4 |  |

**Sagarra-Romero et al. [37]**

|  | Baseline | Postintervention | p-value |
| --- | --- | --- | --- |
| NK (%) |  |  | Between-group p-value not reported |
| Exercise | 6.6± 0.8 | 6.1± 0.9 | 0.06 |
| Control | 6.8± 1.3 | 6.0± 1.1 | 0.46 |

**Schmidt et al. [45]**

| CD16/56 | Baseline | Postintervention | p value |
| --- | --- | --- | --- |
| Resistance exercise | 230.14 (118.26) | 177.48 (118.05) | 0.53 |
| Endurance exercise | 182.65 (82.44) | 109.30 (42.30) | 0.001 |
| Non-exercise | 188.76 (79.30) | 152.16 (99.20) | 0.05 |

**Toffoli et al. [43]**

|  | Baseline | Postintervention | P value |
| --- | --- | --- | --- |
| Relative percentage of cytotoxicity |  |  |  |
| Exercise | 41.5 (28.1) | 49.1 (21.1) | 0.23 |
| Control | 40.8 (28.7) | 35.0 (31.5) |  |
